# A Short Term Test for Carcinogenicity

**DOI:** 10.1038/bjc.1963.9

**Published:** 1963-03

**Authors:** P. M. Peacock, Elizabeth Dick


### A SHORT TERM TEST FOR CARCINOGENICITY

### Mouse Embryo Tissue Homografts in BALB/c Strain Mice

P. M. PEACOCK and ELIZABETH DICK

*From the Cancer Research Department, Royal Beatson Memorial Hospital, Glasgow*

Received for publication November 29, 1962

Rous and Smith (1945) drew attention to the fact that embryo skin of BALB/c strain mice if minced and inoculated into the thigh muscle of adults of the same strain would survive and differentiate, and that by including methylcholanthrene in the inoculum, squamous carcinomas could be induced in the implantation cysts. The same procedure failed to yield such clear-cut results using other strains of mice.

Our procedure for short term testing of substances for carcinogenic potential derived from these observations, is described below. It is essentially simple and we feel it is worth mentioning certain manipulations which permit a rapid turnover of material. Our experiments also confirm that " c " mice are the most satisfactory to use.

### MATERIAL AND METHODS

*Source of implant material*

BALB/c strain female mice were segregated into individual boxes when obviously pregnant, and when within 48 hours of term were killed by cervical dislocation.

*Collection of embryo implant material*

The uterine horns were removed aseptically to a sterile petri dish, where the foetuses were removed from the amniotic sacs, separated from the placentae and transferred to a second dry sterile covered dish.

The foetuses were pinned out one at a time on clean sheets of paper on a cork mat and dissected under low power magnification. The individual tissues were removed aseptically, placed in labelled petri dishes and kept moist with sterile saline. Implants were usually made within 1 hour of the foetal dissections.

*Types of embryo implant material*

Routinely skin, lung (usually right lower lobe, to avoid trachea and oesophagus), stomach and urinary bladder were easily removed and when implanted yielded 100 per cent of growing implants after 16 weeks. Implants of kidney, adrenal, thymus, spleen were sometimes successful, but liver and brain failed to grow.

*Host animals*

Eight-week-old BALB/c mice of either sex were used as hosts for the embryo implants. This age was chosen because the mice were well past weaning and almost of adult size, which simplified surgical procedures.

As we arbitrarily chose 16 weeks for the duration of the tests, the mice were only 6 months old at death and unlikely to have spontaneous tumours.

*Implantation technique*

The outer aspect of the hind legs was shaved using electric clippers. Under Trilene anaesthesia (Trichlorethylene, B.P., manufactured by I.C.I.) a small skin incision was made, posterior to the line of the femur. With a small pair of sharp-pointed scissors the muscle fibres were separated deeply by stabbing in and opening the points. If this was done too close to the bone the femoral vessels were damaged with much bleeding. The implant was placed deeply in the thigh muscle which was allowed to close over it. (This is essential if it is to be retained in position, otherwise there is a tendency for the implant to work its way to a subcutaneous position whence it may escape through the skin wound.)

The skin was closed with a single stitch, taking care to avoid stitching muscle, which would excite a foreign body reaction.

Where a substance was to be tested the material in the solid state was lightly touched by the surface of the implant so that a very small quantity adhered to it. The two were then implanted in close apposition. By using this method we estiated in one of our series, using polycyclic aromatic hydrocarbons, that the quantities tested weighed less than 150 $\mu$g. Substances for test were given code numbers and the identity of the substance was not known at the time of test inoculation.

A different type of tissue was put into each leg, to avoid possible errors at a later date. Details were recorded as follows :

| Date of implant | Mouse No. | Date born | Date weaned | Sex | (L) Leg tissue | (R) Leg tissue | Code number |
|---|---|---|---|---|---|---|---|

*Post operative treatment*

The mice were kept under standard animal house conditions, and examined weekly for the development of nodules. In practice, the presence or absence of a nodule was found to be of no value in the prediction of the development of a malignant lesion, usually being due to the retention of keratin within a cyst.

*Post mortem procedure*

Sixteen weeks after implantation the mice were killed with ether, the skin was reflected from the hind legs, which were then amputated at the hip joint. If the substance under test is fluorescent its persistence can be checked under an ultraviolet lamp, and identified by fluorescence spectrography. We found evidence of both carcinogens and non-carcinogens at death in this way.

*Processing procedure*

Tissues were fixed in 10 per cent formol saline for 2 days, then decalcified in 0·5 per cent formic acid and cation exchange resin (Zeo Korb 225 cation exchange resin, manufactured by Permutit Co.) mixture for 5–7 days. The legs were then bisected in the sagittal plane, which often exposed the implant, and processed in the usual routine manner.

Sections were cut at 8 $\mu$, every 10th one being preserved till the implant appeared in the sections, when 3 to 4 sections at the same interval, containing the implant, were stained and examined before cutting deeper. This permitted serial sections to be taken, if desired, of the remainder of the implant. Routinely only H. and E. sections were examined.

### CONCLUSIONS

By this simple one-stage technique it was possible to expose several types of embryo tissue to the action of a suspect substance during a given period of time, thus widening the scope of the test sequence. As has been shown by one of us in another paper (Peacock, 1962), results comparing very favourably with those from other test methods were obtained in the short space of 16 weeks.

In the light of the experience gained with certain polycyclic aromatic hydrocarbons we feel that perhaps a given test series might be better divided into two groups, one killed after 10 to 12 weeks, and the other after 18 to 20 weeks, if the first gave negative or equivocal results. Anything more prolonged than this would in our view no longer be a short term test, although the results could be of interest and would not invalidate the technique.

### SUMMARY

A simple short term test method of investigating potential carcinogens is described. The procedures of each stage are given in detail.

Results are obtained in 16 weeks using very small quantities of test substance.

This investigation was supported by a full time Research Grant from the British Empire Cancer Campaign.

### REFERENCES

Peacock, P. M.—(1962) *Brit. J. Cancer*, **16**, 701.
Rous, P. and Smith, W. E.—(1945) *J. exp. Med.*, **81**, 597.


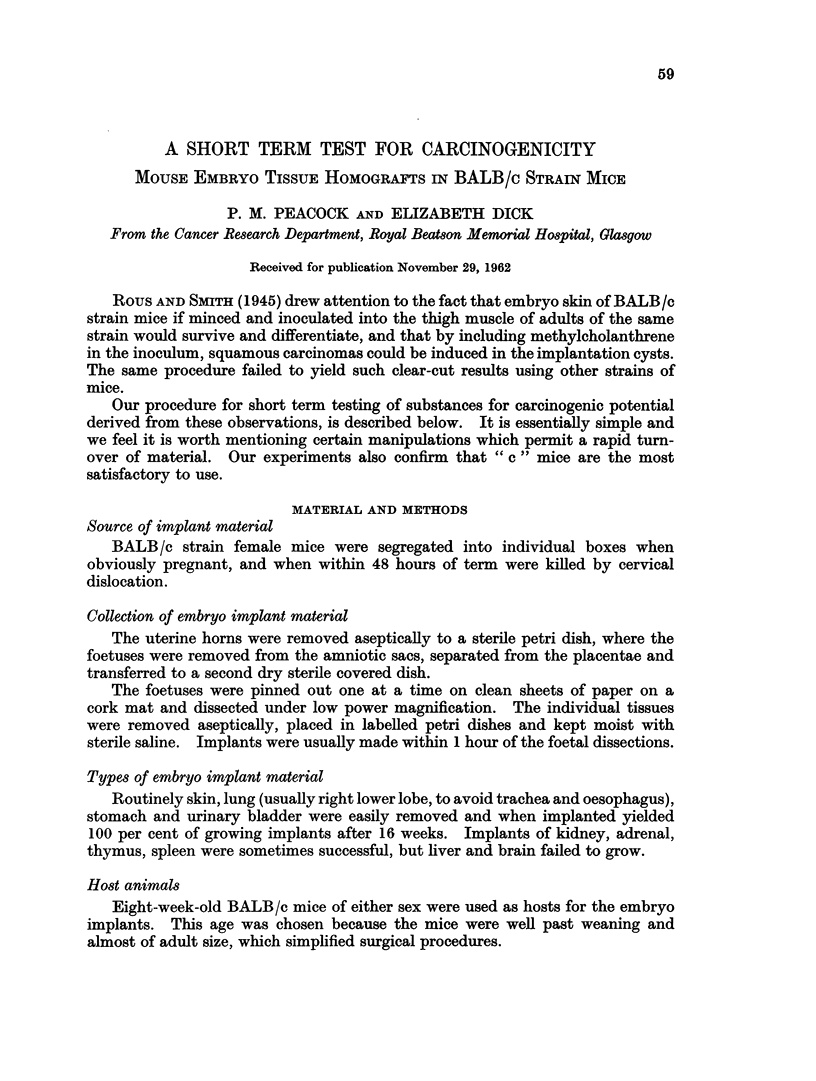

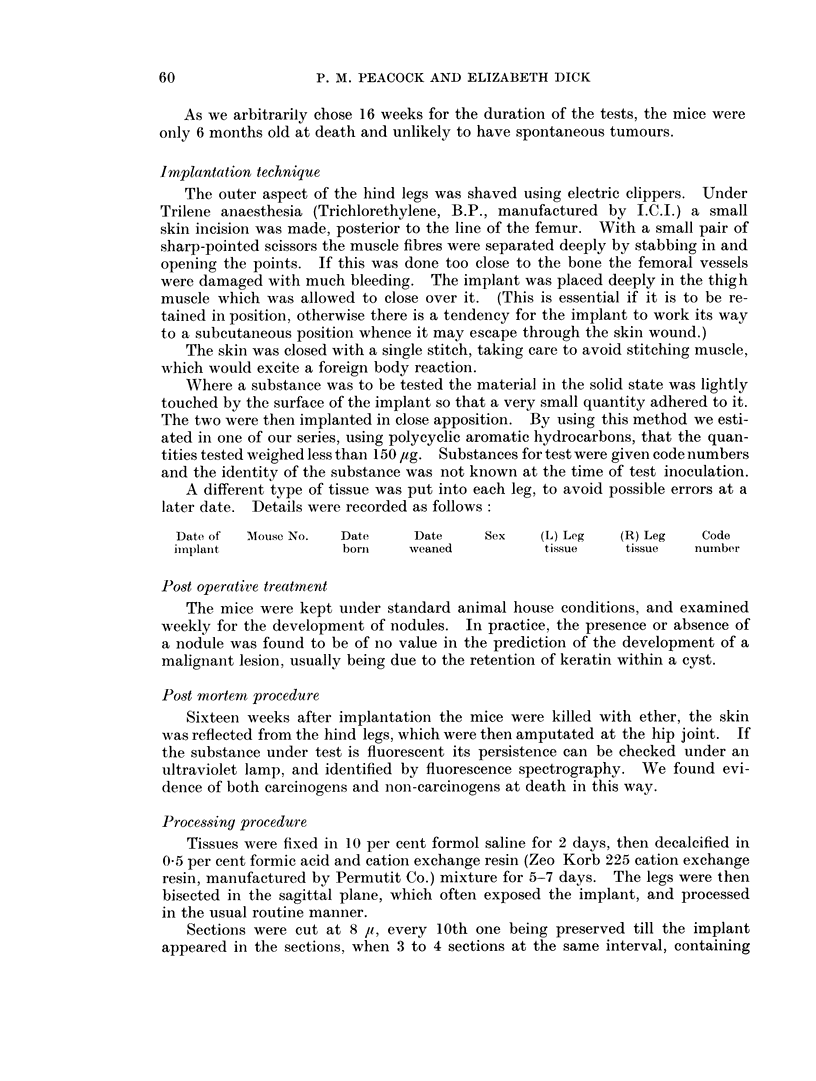

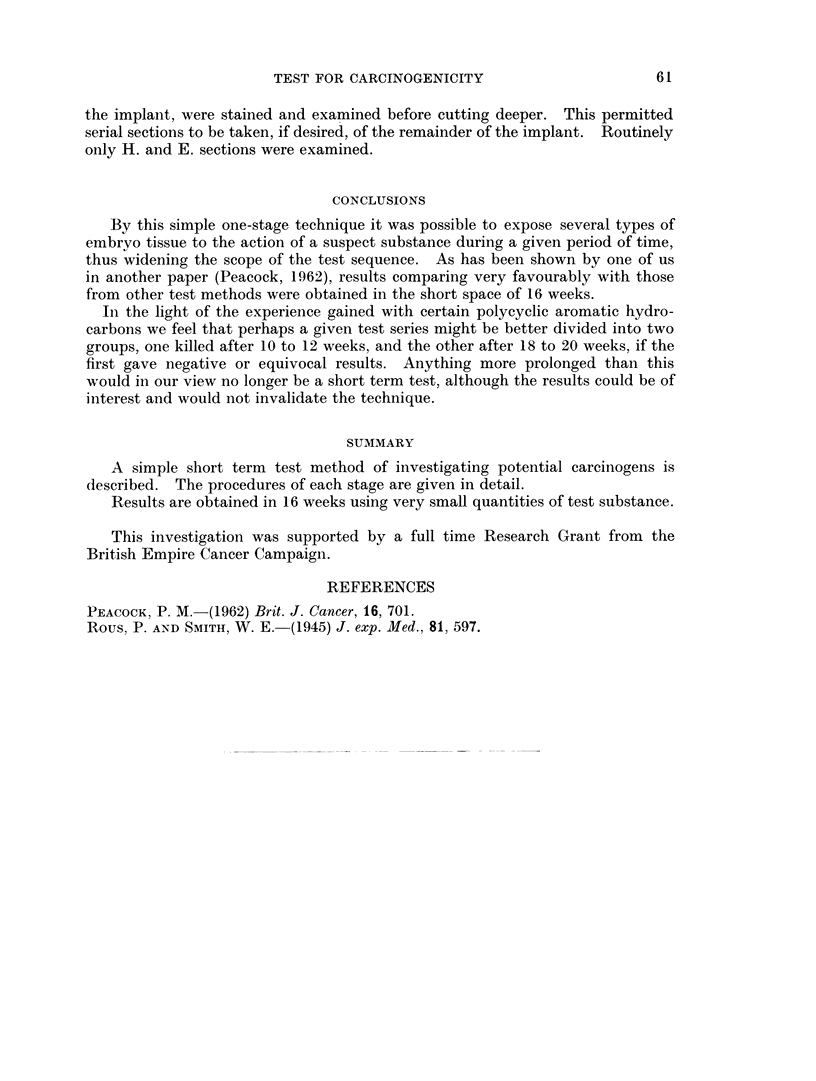


## References

[OCR_00152] PEACOCK P. M. (1962). A short term test for carcinogenicity. The effects of certain closely-related polycyclic aromatic hydrocarbons on embryo tissue homografts in BALB/C strain mice.. Br J Cancer.

